# Multimorbidity - not just an older person's issue. Results from an Australian biomedical study

**DOI:** 10.1186/1471-2458-10-718

**Published:** 2010-11-22

**Authors:** Anne W Taylor, Kay Price, Tiffany K Gill, Robert Adams, Rhiannon Pilkington, Natalie Carrangis, Zumin Shi, David Wilson

**Affiliations:** 1 Population Research & Outcome Studies, South Australian Department of Health, Adelaide, Australia; 2 Department of Medicine, University of Adelaide, Adelaide, Australia; 3 School of Nursing & Midwifery, University of South Australia, Adelaide, Australia; 4 Health Observatory, Queen Elizabeth Hospital, University of Adelaide, Adelaide, Australia; 5 Health Promotion, South Australian Department of Health, Adelaide, Australia

## Abstract

**Background:**

Multimorbidity, the simultaneous occurrence of two or more chronic conditions, is usually associated with older persons. This research assessed multimorbidity across a range of ages so that planners are informed and appropriate prevention programs, management strategies and health service/health care planning can be implemented.

**Methods:**

Multimorbidity was assessed across three age groups from data collected in a major biomedical cohort study (North West Adelaide Health Study). Using randomly selected adults, diabetes, asthma, and chronic obstructive pulmonary disease were determined clinically and cardio-vascular disease, osteoporosis, arthritis and mental health by self-report (ever been told by a doctor). A range of demographic, social, risk and protective factors including high blood pressure and high cholesterol (assessed bio-medically), health service use, quality of life and medication use (linked to government records) were included in the multivariate modelling.

**Results:**

Overall 4.4% of the 20-39 year age group, 15.0% of the 40-59 age group and 39.2% of those aged 60 years of age or older had multimorbidity (17.1% of the total). Of those with multimorbidity, 42.1% were aged less than 60 years of age. A variety of variables were included in the final logistic regression models for the three age groups including family structure, marital status, education attainment, country of birth, smoking status, obesity measurements, medication use, health service utilisation and overall health status.

**Conclusions:**

Multimorbidity is not just associated with older persons and flexible care management support systems, appropriate guidelines and care-coordination programs are required across a broader age range. Issues such as health literacy and polypharamacy are also important considerations. Future research is required into assessing multimorbidity across the life course, prevention of complications and assessment of appropriate self-care strategies.

## Background

Persons with multimorbidity, the simultaneous occurrence of two or more chronic conditions [1,2] are at increased risk of less than best practice care [3,4]. They tend to have more frequent and longer hospitalisations [1,5], greater use of polypharmacy (causing adverse drug effects) [3,6], spend more on their health [4,7,8] and use a greater range of other health care services [3,4]. As the number of health professionals involved in the patients' care increases, patients are more likely to be faced with conflicting instructions and care pathways, and fragmented medical care, making it more challenging for them to adhere to and piece together these instructions, in turn preventing them from participating effectively in their own care [3,4,7,9,10]. The breadth of conflicting advice ranges from treatment, management and medication and includes exercise and diet [9]. Compounding these issues is the problem associated with seeking meaningful information focusing on the actual combination of chronic conditions experienced by the individual.

Prevalence estimates for multimorbidity range from 35% to 80% [2,10-15]. These prevalence estimates vary depending on the method of data collection [1], definition of chronic conditions [16], definition of multimorbidity [12,14,16-18] and the number of chronic conditions included in the analysis [2,15,16]. Many studies have been limited in focus to older persons or have produced results that indicate that multimorbidity is an older persons issue [1,4,11-14], although the broadening of the age ranges is called for [16]. Notwithstanding, due to the ageing population and increased longevity, the prevalence of multimorbidity is expected to increase. It is imperative in the current climate of limited health resources, to move beyond focusing on single chronic conditions and older persons only, to a focus on multimorbidity across all age groups [1,16]. There are important implications for public health and health services/health care planning, especially in regard to the call for increased emphasis on primary care and self management.

Research conducted on these issues in Australia are limited [15,19], yet there is an increasing need to understand the patterns of care and complexity associated with multiple chronic conditions, and to understand the disease combinations/clusters. Of major concern is the increased costs for both the individual and the health care system and how these costs can be contained [7,8]. This study used cross-sectional data from an existing Australian cohort study to examine characteristics of those with multiple chronic conditions, and identify patterns including those associated with health service usage and costs among those with multiple chronic conditions, with a focus on the broad age range of participants. We hypothesise that multimorbidity is important across all age-groups with different characteristics within each age group.

## Method

Data were obtained from the North West Adelaide Health Study (NWAHS), a population based biomedical cohort study established in 2000 [20,21]. This study involves people living in the north-west region of Adelaide, South Australia and covers approximately one third of the South Australian population including a broad range of socioeconomic areas. Its purpose is to investigate the prevalence of chronic conditions and associated health related risk factors, and to monitor progression of diseases over time in order to help plan health care provision in South Australia. Stage 1 was conducted between January 2000 and July 2003 with households randomly selected via the electronic white pages. The last person in the household to have had their birthday and be 18 years of age or older was interviewed over the telephone and invited to attend a clinic for baseline clinical assessment. Of the initial sample in stage 1 (n = 10096), 18.6% were ineligible due to non-connected number, non-residential numbers or fax/modem connections. Of the eligible sample (n = 8213), 2.6% (n = 215) could not be contacted and 26.2% (n = 2148) refused an interview. Of those that were interviewed (n = 5850), 4060 attended the clinic, resulting in a participation rate of 69.4%.

Stage 2 was conducted between May 2004 and February 2006. This stage included a Computer Assisted Telephone Interview (CATI), a self completed questionnaire and a biomedical examination at a clinic. Participants completed all or a combination of these assessments, depending on their capabilities and availability. For Stage 2 the eligible cohort of 4060 was reduced to 3957 (due to deaths). A further 5.9% (n = 233) were unable to be contacted and 4.3% (n = 160) provided no further information, resulting in a response rate of 90.1% (n = 3564). Of the participants that were interviewed and/or completed a questionnaire, 81.0% of the eligible sample (n = 3206) attended a clinic appointment. This analysis is limited to Stage 2 of the study. Detailed analysis addressing bias associated with non-representativeness of the study has previously been published [22].

The chronic conditions used to determine whether a participant had multimorbidity were asthma, cardiovascular disease (CVD), chronic obstructive pulmonary disease (COPD), diabetes, a current mental health condition, arthritis and osteoporosis. A person was deemed to have diabetes if they self-reported having ever been told by a doctor they had the condition or by measurement at the clinic (FPG > = 7.0 mmol/L) [23]. Participants were tested for COPD and asthma at the clinic visit where COPD was determined if FEV1/FVC < 70% and asthma if FEV1 > = 12% & > 200 ml or absolute change greater or equal to 400 ml from pre salbutamol to post salbutamol [24,25]. Participants self-reported if they had ever been told by a doctor they had arthritis, CVD (heart attack, stroke, angina), osteoporosis or a mental health condition (anxiety, depression, stress related problem, other mental health problem). Multimorbidity was defined as having two or more of these seven conditions [2,16].

Demographic data included age, sex, education, annual household income, family structure, marital status, work status and country of birth. Data were collected on self-reported risk factors (smoking status, physical activity level, alcohol risk and depressive symptoms in the last week using CES-D [26]) and clinically measured risk factors (blood pressure, cholesterol, body mass index, waist circumference, waist/hip ratio) using standardised measurements techniques [27-29]. In addition, self-reported data were collected on health service utilisation and health-related quality of life (SF36). All medications that participants were taking, including complementary and alternative medicines, were recorded at the clinic visit. Medicare Benefits Schedule (MBS) records (which includes all services subsidised by the Australian government under the universal health insurance program) and Pharmaceutical Benefits Scheme (PBS) records (which ensures prescription medicines are available at affordable prices to all Australian residents) were obtained after gaining consent from the participant.

The data were weighted by age group, sex, region and probability of selection in the household, to ensure that the sample was representative of the population in the northern and western regions of Adelaide. Descriptive analyses were conducted with SPSS (Version 15.0, SPSS, Chicago, IL, USA).

Univariate odds ratios (OR) were undertaken by age group using STATA V11.0 to examine the association between participants with multiple chronic conditions and a range of related variables. Variables that were statistically significant at the p = 0.25 univariate level [30] were entered into multivariate logistic regression analyses for each age group, to examine the variables independently associated with multiple chronic conditions. In the case of the number of medications taken, the separate variables for the number of prescribed and number of complementary medications were used in preference to the overall number of medications variable. Nonsignificant variables (p value greater than or equal to 0.05) were subsequently omitted in the modelling process until a satisfactory model was obtained. The Hosmer and Lemeshow Goodness of Fit test was adopted as an indicator of the fit of the model; a non-significant value indicated that the model was a good fit for the data [30]. No adjustments in the final models were made. Tests for collinearity were undertaken.

Ethics approval was obtained from the Central Northern Adelaide Health Service Ethics of Human Research Committee.

## Results

The prevalence of multimorbidity was 17.1% (95% CI 15.8 -18.4). Table 1 highlights the prevalence of multimorbidity by age group with 4.4% (95% CI 3.4-5.7) of 20-39 year olds, 15.0% (95% CI 13.1-17.2) of 40-59 year olds and 39.2% (95% CI 35.942.6) of those aged 60 years or older having two or more chronic conditions. Of the 17.1% with multiple chronic conditions, 69.1% (95% CI 65.1 -72.8) had two chronic conditions, 22.9% (95% CI 19.6 -26.6) had three chronic conditions and 8.0% (95% CI 6.0 -10.6) had four or more chronic conditions. Of those with multimorbidity, 57.9% (95% CI 53.8 -62.0) are in the older age group with 42.1% (95% CI 38.0 46.2) aged less than 60 years of age.

**Table 1:** Prevalence of chronic conditions for NWAHS Stage 2 by age group

Number of chronic conditions	Aged 20 - 39		Aged 40 - 59		Aged 60+		Total	
	**n**	**%**	**n**	**%**	**n**	**%**	**n**	**%**
None	837	68.3	622	53.1	191	23.6	1651	51.5
One	334	27.3	373	31.9	300	37.1	1008	31.4
Two	51	4.2	127	10.9	200	24.7	378	11.8
Three	3	0.3	38	3.3	84	10.4	125	3.9
Four or more	-	-	10	0.9	33	4.1	44	1.4
**Total**	**1226**	**100.0**	**1171**	**100.0**	**808**	**100.0**	**3206**	**100.0**
**Total multimorbidity**	**55**	**10.0**	**176**	**32.1**	**317**	**57.9**	**547**	**17.1**
								
	**M**	**SD**	**M**	**SD**	**M**	**SD**	**M**	**SD**
Average number of chronic conditions	0.36*	0.58	0.67*	0.86	1.35*	1.09	**0.72**	**0.92**

* Mean difference is significant between all other age groups at the 0.05 level

Table 2 details the combination of chronic diseases with 24.5% (95% CI 21.2-28.4) of those with multimorbidity having arthritis and a mental health condition and 22.9% (95% CI 19.6-26.6) having arthritis and asthma. This equates to 4.3% (95% CI 3.65.0) and 4.0% (95% CI 3.3-4.7) of the total sample. When analysed by age group, the main clusters were asthma and a mental health condition for 20-39 year olds (67.1%; 95% CI 53.9 -78.1), arthritis and mental health for 40-59 year olds (38.2%; 95% CI 31.3 -45.6) and arthritis and asthma for 60+ year olds (25.2%; 95% CI 20.7 -30.2). Univariate analysis demonstrated that multimorbidity was statistically significantly higher for a range of demographic (Table 3), risk factor (Table 4) and health related variables (Table 5) for each age group.

**Table 2:** Combination of chronic conditions for respondents with multimorbidity

	Respondents with multimorbidity		Overall percent of total sample
	**n**	**%**	**%**
Arthritis and Mental health condition	134	24.6	4.3
Arthritis and Asthma	125	22.9	4.0
Asthma and Mental health condition	98	18.1	3.1
Arthritis and Diabetes	83	15.4	2.7
Arthritis and CVD	80	14.8	2.6
Asthma and COPD	72	13.4	2.3
Arthritis and Osteoporosis	64	11.8	2.0
CVD and Diabetes	49	9.0	1.6
Diabetes and Mental health condition	45	8.3	1.4
Arthritis and COPD	44	8.3	1.4
Asthma and Diabetes	43	7.8	1.3
Asthma and CVD	36	6.7	1.2
Asthma and Osteoporosis	25	4.5	0.8
CVD and Mental health condition	24	4.5	0.8
COPD and Mental health condition	23	4.4	0.8
COPD and CVD	21	4.0	0.7
Mental health condition and Osteoporosis	21	3.8	0.7
COPD and Diabetes	20	3.8	0.7
CVD and Osteoporosis	14	2.5	0.4
COPD and Osteoporosis	13	2.4	0.4
Diabetes and Osteoporosis	6	1.2	0.2

**Table 3:** Univariate analysis of demographic variables associated with participants with multiple chronic conditions compared to those without multimorbidity by age group

	Aged 20 - 39		Aged 40 - 59		Aged 60+	
	**OR (CI 95%)**		**OR (CI 95%)**		**OR (CI 95%)**	
	N = 1226		N = 1171		N = 808	
**Sex**						
Male	1.00		1.00		1.00	
Female	1.18 (0.48 - 2.86)		1.19 (0.86 - 1.63)		1.23 (0.96 - 1.58)	
Highest education level obtained*						
Bachelor degree or higher	1.00		1.00		1.00	
Secondary	***5.16 ***(1.41 - 18.83)		1.47 (0.86 - 2.50)		***2.99 ***(1.38 - 6.46)	
Trade/Certificate/Diploma	2.30 (0.58 - 9.19)		1.17 (0.68 - 2.03)		***2.25 ***(1.02 - 4.94)	
**Gross annual household income**						
More than $80,001	1.00		1.00		1.00	
$20,001-80,000	1.70 (0.54 - 5.32)		1.39 (0.83 - 2.33)		1.29 (0.55 - 3.02)	
Up to $20,000	0.87 (0.19 - 4.08)		***5.87 ***(3.35 - 10.29)		1.64 (0.70 - 3.83)	
Not stated	-		***2.63 ***(1.05 - 6.59)		1.52 (0.59 - 3.89)	
Family Structure*						
Family & Children	1.00		1.00		1.00	
Adult living with partner	2.12 (0.71 - 6.36)		***2.31 ***(1.50 - 3.56)		***1.95 ***(1.05 - 3.60)	
Adult living alone	1.33 (0.35 - 5.06)		***4.69 ***(2.95 - 7.46)		***2.45 ***(1.31 - 4.58)	
Adults-related/unrelated	-		1.58 (0.85 - 2.95)		***2.47 ***(1.21 - 5.04)	
Other	0.66 (0.21 - 2.07)		1.59 (0.93 - 2.72)		***2.37 ***(1.13 - 4.98)	
Martial Status*						
Never married	1.00		1.00		1.00	
Married or living with partner	1.15 (0.43 - 3.10)		0.83 (0.48 - 1.44)		0.58 (0.28 - 1.24)	
Separated/divorced/widowed	0.78 (0.14 - 4.37)		***1.91 ***(1.05 - 3.45)		0.76 (0.36 - 1.64)	
Work Status*						
Full time employed	1.00		1.00		1.00	
Part time/casual	1.04 (0.36 - 3.04)		***1.66 ***(1.09 - 2.53)		1.83 (0.77 - 4.38)	
Economically inactive	0.82 (0.28 - 2.43)		***3.43 ***(2.36 - 4.98)		***3.98 ***(2.05 - 7.73)	
**Country of birth**						
Australia	1.00		1.00		1.00	
UK/Ireland	2.67 (0.86 - 8.33)		1.25 (0.83 - 1.88)		0.92 (0.68 - 1.23)	
Europe	3.70 (0.45 - 30.40)		1.06 (0.60 - 1.86)		0.71 (0.48 - 1.04)	
Asia/other	3.86 (0.47 - 31.57)		1.30 (0.55 - 3.06)		***0.23 ***(0.08 - 0.64)	

Other/not stated removed from analysis

Note: ***Italics and bold ***indicate significance at the 0.05 level

**Table 4:** Univariate analysis of risk factor variables associated with participants with multiple chronic conditions compared to those without multimorbidity by age group

	Aged 20 - 39		Aged 40 - 59		Aged 60+	
	**OR (CI 95%)**		**OR (CI 95%)**		**OR (CI 95%)**	
	N = 1226		N = 1171		N = 808	
Smoking*						
Non-smoker	1.00		1.00		1.00	
Ex-smoker	0.99 (0.32 - 3.13)		1.39 (0.96 - 2.02)		***1.31 ***(1.01 - 1.71)	
Current smoker	1.80 (0.66 - 4.89)		***1.80 ***(1.20 - 2.70)		1.14 (0.71 - 1.84)	
Physical Activity*						
Undertakes exercise (low/med/high)	1.00		1.00		1.00	
Sedentary	2.26 (0.87 - 5.84)		1.22 (0.85 - 1.73)		***1.53 ***(1.15 - 2.03)	
Alcohol Risk*						
Non drinkers/no risk	1.00		1.00		1.00	
Low risk to very high risk	0.76 (0.32 - 1.80)		0.93 (0.67 - 1.28)		0.98 (0.75 - 1.27)	
High Blood Pressure* (≥140/90 mmHg)						
No	1.00		1.00		1.00	
Yes	1.98 (0.64 - 6.08)		1.37 (0.97 - 1.92)		1.17 (0.91 - 1.50)	
High Cholesterol level* (≥ 5.5 mmol/L)						
No	1.00		1.00		1.00	
Yes	1.12 (0.42 - 3.00)		0.86 (0.63 - 1.18)		***0.52 ***(0.40 - 0.68)	
Body Mass Index (BMI)*						
Normal (18.50 - 24.99)	1.00		1.00		1.00	
Underweight (< 18.50)	1.45 (0.14 - 14.69)		0.49 (0.06 - 3.86)		0.46 (0.08 - 2.79)	
Overweight (25.00 - 29.99)	0.98 (0.30 - 3.15)		1.17 (0.75 - 1.82)		1.17 (0.86 - 1.61)	
Obese (30.00+)	***3.11 ***(1.06 - 9.11)		***2.03 ***(1.32 - 3.12)		***1.71 ***(1.22 - 2.39)	
High Waist Circumference*^#^						
No	1.00		1.00		1.00	
Yes	2.54 (0.98 - 6.58)		***2.30 ***(1.53 - 3.47)		***1.60 ***(1.17 - 2.20)	
High Waist Hip Ratio*^##^						
No	1.00		1.00		1.00	
Yes	3.14 (0.99 - 9.98)		***2.92 ***(2.10 - 4.05)		***1.72 ***(1.33 - 2.24)	
Depressive Symptoms*						
No	1.00		1.00		1.00	
Yes	***4.22 ***(1.61 - 11.05)		***4.13 ***(2.81 - 6.07)		***2.47 ***(1.59 - 3.84)	

*Other/not stated removed from analysis

^#^Men ≥ 95 cm, women ≥ 80 cm (no further weight should be gained)

^##^Men > 1.0 men, women > 0.85

Note: ***Italics and bold ***indicate significance at the 0.05 level

**Table 5:** Univariate analysis of health related variables associated with participants with multiple chronic conditions compared to those without multimorbidity by age group

	Aged 20 - 39		Aged 40 - 59		Aged 60+	
	**OR (CI 95%)**		**OR (CI 95%)**		**OR (CI 95%)**	
	N = 1226		N = 1171		N = 808	
Overall health status*						
Excellent	1.00		1.00		1.00	
Very good	0.40 (0.04 - 3.64)		1.92 (0.76 - 4.86)		1.84 (0.76 - 4.44)	
Good	3.23 (0.40 - 25.80)		***4.61 ***(1.89 - 11.23)		***3.88 ***(1.64 - 9.17)	
Fair	7.80 (0.86 - 70.30)		***15.90 ***(6.32 - 39.98)		***7.52 ***(3.12 - 18.17)	
Poor	-		***14.59 ***(4.14 - 51.41)		***15.71 ***(4.32 - 57.15)	
Health service visits*						
None/one	1.00		1.00		1.00	
Two to Four	1.28 (0.46 - 3.57)		***2.10 ***(1.31 - 3.82)		***2.00 ***(1.38 - 2.89)	
Five or more	2.13 (0.56 - 8.15)		***7.28 ***(4.19 - 12.64)		***5.43 ***(3.44 - 8.59)	
**Number of medicines (prescribed & complementary)**						
None/not stated	1.00		1.00		1.00	
One to three	2.21 (0.73 - 6.67)		***3.98 ***(2.13 - 7.41)		***6.09 ***(2.63 - 14.13)	
Four or more	***4.35 ***(1.06 - 17.70)		***14.72 ***(7.95 - 27.24)		***21.53 ***(9.44 - 49.11)	
**Number of prescribed medications**						
None/not stated	1.00		1.00		1.00	
One to three	***3.54 ***(1.19 - 10.55)		***4.69 ***(2.74 - 8.02)		***5.08 ***(2.44 - 10.61)	
Four or more	***7.07 ***(1.39 - 35.99)		***22.45 ***(12.80 - 39.38)		***22.36 ***(10.83 - 46.16)	
**Number of complementary medicines**						
None/not stated	1.00		1.00		1.00	
One to two	0.61 (0.19 - 1.97)		1.39 (0.97 - 2.00)		1.14 (0.85 - 1.53)	
Three or more	1.10 (0.13 - 9.01)		1.54 (0.87 - 2.73)		1.11 (0.67 - 1.84)	
MBS Cost (2004 - 2007)*						
Less than $2000	1.00		1.00		1.00	
$2001 to $6000	***3.17 ***(1.18 - 8.52)		***2.28 ***(1.57 - 3.30)		***2.14 ***(1.54 - 2.97)	
$6001+	***5.57 ***(1.64 - 20.19)		***10.32 ***(6.33 - 16.81)		***3.11 ***(2.17 - 4.48)	
MBS Services (2004 - 2007)*						
Less than 50	1.00		1.00		1.00	
51 to 200	***2.99 ***(1.20 - 7.46)		***3.08 ***(2.14 - 4.44)		***1.95 ***(1.34 - 2.84)	
201+	4.73 (0.50 - 44.46)		***11.62 ***(6.02 - 22.44)		***3.88 ***(2.49 - 6.06)	
PBS Cost (2004 - 2007)*						
Less than $150	1.00		1.00		1.00	
$151 to $1000	1.93 (0.51 - 7.32)		***2.33 ***(1.38 - 3.93)		0.97 (0.53 - 1.75)	
$1001+	3.07 (1.00 - 9.47)		***6.41 ***(4.44 - 9.25)		***2.79 ***(2.01 - 3.87)	
PBS Items processed (2004 - 2007)*						
Less than 25	1.00		1.00		1.00	
26 to 150	***3.12 ***(1.17 - 8.29)		***3.61 ***(2.49 - 5.24)		***1.67 ***(1.17 - 2.39)	
151+	-		***12.61 ***(7.13 - 22.30)		***4.89 ***(3.42 - 6.98)	

*Other/not stated removed from analysis

Note: ***Italics and bold ***indicate significance at the 0.05 level

Multivariate logistic regression analyses (Table 6 and Table 7) were undertaken for each of the three age groups and revealed that those with multiple chronic conditions aged 20 to 39 years (Goodness of Fit X^2 ^= 4.55, p = 0.81) were statistically significantly more likely to be living with a partner, have a secondary level of education, be born in the United Kingdom or Ireland, have a high waist circumference and have MBS costs of $2000 or more. Those aged 40 to 59 years (Goodness of fit X^2 ^= 5.52, p = 0.70) were statistically significantly more likely to be living with a partner or living alone, be separated, divorced or widowed, be a current smoker, have a high waist hip ratio, have depressive symptoms, be using prescribed medicines, have MBS costs of over $6000 and self report that they have a fair overall health status. People aged over 60 years of age (Goodness of fit X^2 ^= 7.73, p = 0.46) were statistically significantly more likely be living alone or with unrelated adults, have a secondary level of education or trade, certificate or diploma, have used five or more health services in the last year, use prescribed medicines, have over 150 PBS items processed in the previous three years, and have fair or poor overall health status. No collinearity was found in any model.

**Table 6:** Multivariate analysis of demographic, risk factor and health related variables associated with participants with multiple chronic conditions compared to those without multimorbidity for respondents, by age group

	Aged 20 - 39	Aged 40 - 59	Aged 60+
	**OR (CI 95%)**	**OR (CI 95%)**	**OR (CI 95%)**
**Family Structure**			
Family & Children	1.00	1.00	1.00
Adult living with partner	***3.61 ***(1.22 - 10.73)	***1.70 ***(1.01 - 2.84)	2.22 (0.92 - 5.25)
Adult living alone	3.49 (0.91 - 13.41)	***3.51 ***(1.41 - 8.74)	***3.19 ***(1.33 - 7.63)
Adults-related/unrelated	-	1.09 (0.45 - 2.67)	***2.91 ***(1.10 - 7.68)
Other	1.05 (0.30 - 3.73)	1.25 (0.60 - 2.60)	2.34 (0.85 - 6.43)
**Martial Status**			
Never married		1.00	
Married or living with partner		2.24 (0.83 - 6.04)	
Separated/divorced/widowed		***2.35 ***(1.09 - 5.03)	
**Highest education level obtained**			
Bachelor degree or higher	1.00		1.00
Secondary	***5.08 ***(1.29 - 19.92)		***3.13 ***(1.31 - 7.46)
Trade/Certificate/Diploma	1.86 (0.43 - 7.96)		***2.85 ***(1.18 - 6.89)
**Country of birth**			
Australia	1.00		
UK/Ireland	***6.65 ***(1.72 - 25.64)		
Europe	4.55 (0.77 - 26.71)		
Asia/other	-		
**Smoking**			
Non-smoker		1.00	
Ex-smoker		1.33 (0.85 - 2.07)	
Current smoker		***1.71 ***(1.02 - 2.86)	
**High Waist Circumference**			
No	1.00		
Yes	***3.60 ***(1.34 - 9.63)		
**High Waist Hip Ratio**			
No		1.00	
Yes		***1.70 ***(1.14 - 2.54)	

Note: ***Italics and bold ***indicate significance at the 0.05 level

**Table 7:** Multivariate analysis of demographic, risk factor and health related variables associated with participants with multiple chronic conditions for respondents compared to those without multimorbidity, by age group (continued)

	Aged 20 - 39	Aged 40 - 59	Aged 60+
	**OR (CI 95%)**	**OR (CI 95%)**	**OR (CI 95%)**
**Depressive Symptoms**			
No		1.00	
Yes		***2.09 ***(1.26 - 3.46)	
**Health Service Visits**			
None/one			1.00
Two to Four			1.22 (0.77 - 1.92)
Five or more			***2.27 ***(1.28 - 4.02)
**Number of prescribed medications**			
None/not stated		1.00	1.00
One to three		***3.47 ***(1.89 - 6.39)	***3.91 ***(1.66 - 9.18)
Four or more		***10.73 ***(5.24 - 21.94)	***10.95 ***(4.52 - 26.52)
**MBS Cost (2004 - 2007)**			
Less than $2000	1.00	1.00	
$2001 to $6000	***3.84 ***(1.29 - 11.43)	1.15 (0.73 - 1.82)	
$6001+	***7.88 ***(1.86 - 33.46)	***2.84 ***(1.57 - 5.13)	
**PBS Items processed (2004 - 2007)**			
Less than 25			1.00
26 to 150			1.40 (0.92 - 2.13)
151+			***1.80 ***(1.14 - 2.85)
**Overall health status**			
Excellent		1.00	1.00
Very good		1.36 (0.50 - 3.70)	1.24 (0.50 - 3.09)
Good		2.27 (0.86 - 5.97)	1.80 (0.74 - 4.40)
Fair		***3.75 ***(1.29 - 10.85)	***3.22 ***(1.28 - 8.12)
Poor		3.59 (0.86 - 14.91)	***15.58 ***(2.80 - 86.75)

Note: ***Italics and bold ***indicate significance at the 0.05 level

## Discussion

This analysis has used data from a randomly selected sample of community living Australians to describe, by age group, the demographic, risk factor, health service utilisation and quality of life of people living with more than one chronic condition. The results indicate that the profile of people living with multimorbidity is different for each age group but on the whole these people are more likely to take medications, spend more on their health and have a lower quality of life. There was no difference by gender which was also observed in a previous Australian study [15].

The overall prevalence of multimorbidity of 17.1% is lower than previous studies due to the population-wide, random nature of the sample and the limited total number of chronic conditions assessed. Previous studies have used a variety of non-random, or non-population wide samples or used specific older populations [2,4,11,12,14]. Results from our study support the finding that the prevalence of multimorbidity increases with age. Although Smith and Dourd [1] argue that multimorbidity is a "normal state of affairs" for those aged over 65 years of age, this study has highlighted that multimorbidity is also a problem in younger age groups with over 40% of those having multimorbidity aged less than 60 years of age. Mercer et al [16] call for more research of multimorbidity across the life course for prevention and early intervention purposes but also because younger people fail to qualify for the typical geriatric care services that seem to be the focus for multiple chronic conditions.

Polypharmacy was an issue for both the 40-59 and 60+ age groups with both groups over ten times more likely to be taking four-or-more prescribed medications on a daily basis, in turn increasing the risk of adverse drug effects and increasing health care costs to the individual [13,31]. This research has no relevant data on adverse outcomes or mortality often associated with polypharmacy [6]. Medication costs are a cause for concern for health systems as well as for the person with the conditions [9]. In this study all groups were associated with increasing health care costs - MBS for younger people, prescribed medication and MBS for the 40-59 year olds and prescribed medications and PBS items for the older groups.

Of particular significance is the fact that 40-59 year olds with multimorbidity are 1.71 times more likely to be current smokers than non-smokers. One has to ask if these people with multiple chronic conditions are smoking because of their health status or was smoking the cause of their multimorbidity. This needs to be further investigated and appropriate promotion activities targeted. Previous studies have linked smoking and mental health conditions suggesting that cigarette smoking might ease anxiety and depression [32]. However, it must also be highlighted that smokers have a greater risk for depression which increases with the severity of nicotine dependence. Therefore, smoking status and the likelihood of depressive symptoms in those with multimorbidity is strongly inter-related.

Results of the multivariate analyses indicate that participants aged 20-39 years with lower education levels were statistically significantly more likely to have multiple morbidities compared to those with higher education levels. Although previous studies have shown this relationship [11], it is normally within older age groups whose education opportunities have historically been limited although in this age group it could be that formal education is still being undertaken and, as such, the educational level may be an underestimation.

Obesity, in terms of high waist circumference for 20-39 year olds and waist hip ratio for 40-59 year olds has also previously been shown to be linked with multimorbidity [11,32] with rates of obesity increasing in line with the number of chronic conditions. The major public health emphasis on healthy weight should have rebound effects on the prevalence of multimorbidity in the future.

One of the major strengths of this study is the use of a large randomly selected sample of the South Australian population. The large sample size allows for greater generalisation of results. Clinically accessed information on chronic conditions also contributes to the strength of this study. Combining this with data collected on a wide range of risk factors and socio-demographic variables allows for a rich data source which can be analysed by various means. Linking participants MBS and PBS data provides a further insight into health care utilisation and cost, highlighting which chronic conditions or combinations of chronic conditions require significant care and in turn how costly this care can be. The weaknesses of this study include the cross-sectional nature of the data collection with the consequent inability to determine direction of effect. In addition, the reliance on self-report for some of the assessed variables is vulnerable to social desirability or other biased responses.

We recognise that there are limitations of this study, particularly the shortcoming associated with self-reported data collection. In addition, sampling by telephone directory is likely to under sample some groups in the community. The study also only involves community living adults and as such people living in supported accommodation such as aged care facilities were, on the whole, not included although 'home' visits to the new place of residence was undertaken if acceptable and appropriate. Detailed analysis of the bias of the NWAHS sample caused both by telephone sampling and self-reported measures has been assessed and published [22]. Another limitation of the study is that not all chronic conditions (i.e. cancer, deafness, sight problems, dementia) were included in the definition of what was reported as a chronic condition. Other studies, especially those using clinical notes or administrative databases, have used the full list of ICD codes and hence our study, limiting itself to seven chronic conditions, will be an underestimation of the true prevalence of multimorbidity in the Australian society. A further weakness of the study was the fact that no indicator of levels of severity such as the Cumulative Illness Rating Scale, CIRS) [2], the Index of Coexisting Disease, the Kaplan Index, or Charlson Index [18] were used. All chronic conditions were treated equally when in fact one or more of the chronic conditions could have a more dominant effect. A further adaptation to the study design could be undertaken by incorporating conditions such as hypertension, hypercholesterolemia, and obesity as chronic conditions rather than risk factors, as described by others [14,15].

Further research is required in this relatively new field, especially in regard to interventions to improve outcomes for people with multimorbidity, to determine the relationship between multimorbidity and shared risk factors [33], to access the effect of multimorbidity across the life course, to investigate the effect on carers, to prevent complications attributed to multimorbidity [8] and to investigate the barriers and increased difficulties associated with self-care [16,34]. Developing appropriate self-management strategies for people with clusters of chronic diseases are important.

## Conclusion

Preventing, treating and managing chronic conditions is complex but is one of the major challenges facing the health care systems. This research has identified a range of factors associated with multimorbidity such as family structure, marital status, education level, country of birth, medication use, health service use, existence of depressive symptoms, smoking status, overall health status, high waist hip ratio and waist circumference which vary according to age group. This valuable information and increased understanding can lead to improved prevention strategies. Important in forward endeavours is the need to access and modify the many clinical guidelines and disease management programs that concentrate on single conditions [3,34]. Flexibility within the shared decisions making strategies is crucial and importance must be placed on the need for effective communication. Multimorbidity often requires flexible care management support systems and care co-ordination programs, and the current challenge is to accommodate these needs in the complex, multidimensional world of multimorbidity.

## Abbreviations

CATI: Computer Assisted Telephone Interviewing; CES-D: Centre for Epidemiologic Studies Depression Scale; CIRS: Cumulative Illness Rating Scale; COPD: Chronic Obstructive Pulmonary Disease; CVD: Cardio Vascular Disease; FEV: Forced expiratory volume in one second; FPG: Fasting plasma glucose; FVC: Forced vital capacity; ICD: International Classification of Diseases; MBS: Medicare Benefits Schedule; NWAHS: North West Adelaide Health Study; PBS: Pharmaceutical Benefits Scheme; SF36: Short Form - 36; SPSS: Statistical Package for Social Sciences

## Competing interests

The authors declare that they have no competing interests.

## Authors' contributions

AT conceived the design of the study, participated in the co-ordination of the study and drafted the manuscript. KP participated in the design and co-ordination of the study and was involved in the drafting of the manuscript. TG participated in the design and co-ordination of the study, performed statistical analyses and was involved in the drafting of the manuscript. RA participated in the design and co-ordination of the study, and was involved in the drafting of the manuscript. RP performed statistical analyses and was involved in the drafting of the manuscript. NC performed statistical analyses and was involved in the drafting of the manuscript. ZS performed statistical analyses and was involved in the drafting of the manuscript. DW conceived the design of the study, participated in the co-ordination of the study, and was involved in the drafting of the manuscript. All authors read and approved the final manuscript.

## Pre-publication history

The pre-publication history for this paper can be accessed here:

http://www.biomedcentral.com/1471-2458/10/718/prepub
